# Post-identifiability in changing sociotechnological genomic data environments

**DOI:** 10.1057/s41292-023-00299-7

**Published:** 2023-03-28

**Authors:** Kaya Akyüz, Melanie Goisauf, Gauthier Chassang, Łukasz Kozera, Signe Mežinska, Olga Tzortzatou-Nanopoulou, Michaela Th. Mayrhofer

**Affiliations:** 1 Department of Science and Technology Studies, University of Vienna, Universitätsstraße 7/Stiege II/6, Stock (NIG), 1010 Vienna, Austria; 2 BBMRI-ERIC, Graz, Austria; 3 CERPOP, Université de Toulouse, Inserm, Université Paul Sabatier, Toulouse, France; 4 Plateforme GenoToul Societal “Ethique et Biosciences”, Toulouse, France; 5 Institute of Clinical and Preventive Medicine, University of Latvia, Riga, Latvia; 6 BBMRI.LV, Riga, Latvia; 7 Biomedical Research Foundation of the Academy of Athens, Athens, Greece; 8 BBMRI.GR, Athens, Greece

**Keywords:** Genomic identifiability, Henrietta Lacks, Post-identifiability, Postgenomics, Privacy, Infrastructures

## Abstract

Data practices in biomedical research often rely on standards that build on normative assumptions regarding privacy and involve ‘ethics work.’ In an increasingly datafied research environment, identifiability gains a new temporal and spatial dimension, especially in regard to genomic data. In this paper, we analyze how genomic identifiability is considered as a specific data issue in a recent controversial case: publication of the genome sequence of the HeLa cell line. Considering developments in the sociotechnological and data environment, such as big data, biomedical, recreational, and research uses of genomics, our analysis highlights what it means to be (re-)identifiable in the postgenomic era. By showing how the risk of genomic identifiability is not a specificity of the HeLa controversy, but rather a systematic data issue, we argue that a new conceptualization is needed. With the notion of *post-identifiability* as a sociotechnological situation, we show how past assumptions and ideas about future possibilities come together in the case of genomic identifiability. We conclude by discussing how kinship, temporality, and openness are subject to renewed negotiations along with the changing understandings and expectations of identifiability and status of genomic data.

## Introduction

The HeLa cell line, generated in 1951 from a sample taken from Henrietta Lacks’ tumor without her consent, has been the first immortalized human cell line around which entire research infrastructures have been built (Landecker 2007). The story of HeLa cannot be separated from the story of Henrietta Lacks, the Lacks family, and institutional racism in science (Reardon 2013; Wald 2012; Landecker 2007). In early years, many assumed the cells came from a fictional white woman, Helen Lane; however, with the obituary for George Gey, the scientist responsible for the development and distribution of the cell line, Henrietta Lacks was named for the first time as the origin of HeLa (Jones et al. 1971). Shortly after, the Lacks family was contacted by scientists for their blood samples (Culliton 1974; Skloot 2010) due to contamination issues that is estimated to have led to tens of thousands of publications produced with HeLa cells misidentified as other cell lines (Horbach and Halffman 2017). In 2013, HeLa was once more at the center of a controversy with the release of the genome sequence for the cell line (Landry et al. 2013) and uproar it caused as the Lacks family members raised concerns regarding the risks this poses in terms of their genomic identifiability (Callaway 2013b). Genomic identifiability as an issue is gaining momentum along with forensic genealogy applications that receive attention in the media, such as the Golden State Killer case (Phillips 2018) and research about the identifiability risks especially regarding publicly accessible genomic databases (Erlich and Narayanan 2014; Erlich et al. 2018; Gymrek et al. 2013).

For the most well-known cell line, the timing of the genome sequence publication may even be considered late. By the time Human Genome Project was completed in 2003, genomes of many model organisms (the yeast *Saccharomyces cerevisiae*, the fruit fly *Drosophila melanogaster*, the mouse *Mus musculus* and the flowering plant *Arabidopsis thaliana*) were already available (Tickle and Urrutia 2017). Such genome sequences of different organisms, including the human genome itself, have been the product of international consortia working to produce a common resource for the research community, producing data according to FAIR data principles (Wilkinson et al. 2016), long before the term FAIR emerged. At a time big data and the use in research as well as applications, such as artificial intelligence are increasingly relevant for science and society and are attracting attention of scholars (Kitchin 2014; Borgman 2015; Crawford 2021; D'Ignazio and Klein 2020), identifiability through genomic data is becoming more salient along with developments in data sharing platforms as well as cloud and next-generation sequencing technologies (Martinez-Martin and Magnus 2019; Bonomi et al. 2020; Narayan 2020; Carter 2019).

In this article, we unpack the genomic identifiability issue through the case of the recent HeLa controversy with a science and technology studies (STS)-informed, interdisciplinary lens. The episode that we discuss gravitates around the publication of the HeLa genome sequence, and in this paper, following a discursive analysis of the case and the construction of genomic identifiability as a risk in relevant guidelines and legal framework, we are showing how the standardized practices of risk management may fall short of being proactive even in well-known cases of controversy. By reconstructing the controversy HeLa 2.0, we aim to analyze how genomic identifiability as risk can be re-considered by posing the following questions: Is HeLa 2.0 a unique case or does it point to a far-reaching systematic issue in the current datafied research environment? What does this controversy tell us about genomic identifiability as a risk from the STS perspective that analyzing the laws and guidelines alone cannot?

## Analytical framing

The analytical perspective of this study is inspired by *sociology of expectations* (Brown and Michael 2003) to unpack the constitutive elements of genomic identifiability in the HeLa 2.0 controversy. Following this approach, the past, present, and future cannot be understood as separate, but interdependent temporalities. Consequently, innovations and applications in health sciences and (bio-)technology cannot be seen as evolving detached from their past and potential futures. Rather, they need to be understood as situated within the dynamics of current and former expectations. To capture the modus operandi of this dynamic, the approach analytically distinguishes between two patterns: how the future was represented in the past and the ways used in the present to construct or engage with the future. Thus, enactments as well as social and materialized practice of ‘retrospecting prospects’ and ‘prospecting retrospects’ allow insights into the dynamics of changing expectations: “the recollection of past futures or how the future was once represented” and “how these prospects are deployed in the real-time now, to construct futures,” respectively (Brown and Michael 2003, p. 4).

Thus, the concept of sociology of expectations enables us to focus our analysis on the discursive practices through which actors, actions, posts in social media, guidelines, policies, regulations as well as other documents assemble and culminate in the HeLa 2.0 controversy on genomic identifiability. In doing so, this analytical approach allows us to draw conclusions beyond the case, as the expectations enacted are embedded in assumptions and developments regarding present futures of data environments.

## Methodology

The empirical research focused on two discursive sites that are constitutive for the issue of genomic identifiability: (1) the analysis of the discourse on the HeLa 2.0 controversy in a public-scientific arena and (2) the analysis of the relevant international guiding documents and legal framework in order to show how identifiability is constructed as a risk. We understand discursive practice as situated social practice, as “sayings” that (besides “doings”) constitute a social phenomenon (Schatzki et al. 2001).

### Case description: HeLa 2.0

We approach the HeLa 2.0 controversy as an empirical case study to carve out constitutive elements that help understanding a broader discourse on genomic identifiability in data-intensive research practice. While case study approach in STS has received critique, it has great potential for contributing to an interdisciplinary research practice (Beaulieu et al. 2007) and bringing multiple perspectives together as in our team of researchers with diverse backgrounds, from law to biomedicine, STS to bioethics.

Sampling of the empirical material followed key topics and actors that retrospectively dominated the HeLa 2.0 controversy. Scholars observe that in the 2000s, HeLa controversy has become embedded in education and societal discourse, especially in relation to bioethics and issues of race. This owes partially due to Skloot’s (2010) bestseller on the topic where the media attention to the book was dominated by “informed consent,” and issues of “welfare of the vulnerable” and “donor compensation” (Nisbet and Fahy 2013, p. 1). In social scientists’ engagement with publics, the controversy was brought up in relation to perceived risks and benefits regarding the use of electronic health data, residual tissue samples (Botkin et al. 2015, p. 208), and genomic biobanking (Cohn et al. 2015) as well as on donations for stem cell research, where the HeLa cell line served to bring immortalization as a problem in light of commercialization and inappropriate use of the cells (Dasgupta et al. 2014, pp. 10–11; Lee et al. 2019). Beskow points out a broad influence of the controversy, claiming it to be a “catalyst for policy change” due to it being embedded in education, popular culture, and the media (2016, p. 396). Against this background, HeLa 2.0 as a case is not merely the continuation of the historical controversy, but an episode from recent history that reveals potential issues that are emerging as the role of human genomic data expands and thus constitutes a case to think with, rather than being a generalizable situation.

For the reconstruction of the HeLa 2.0, we relied on the publicly available resources from the year 2013 which directly referred to the controversy, such as news/commentaries, statements, and articles in academic journals as well as scientists’ blogs and Twitter feeds, newspaper articles, and institutional websites/databases (*n* = 24). In doing so, the objective has been not only to recount what happened in this short period, but also to quote important components of the rhetorical repertoire used by the major actors in the controversy. The selection of the major actors followed a snowball approach through identification of additional actors within the analyzed material starting out with Rebecca Skloot as the popularizer of the controversy, Francis Collins as the Director of the National Institutes of Health (NIH), the authors of the study/researchers who have conducted the research on sequencing of the HeLa line, their institution, and Lacks family members.

### Analysis of the relevant international guiding documents and legal framework

For the second part, we have focused on the selected documents (Table 1) as well as relevant academic literature to analyze how genomic identifiability is (not) constructed as a risk. We selected documents that would be central as legal frameworks and guidelines for those who are involved in production, storage, and sharing of human biological samples and data, based on institutional knowledge of the co-authors who have been working at a hub for biobanks and have been in contact with biobanking community as an interdisciplinary team.

**Table 1:** Selected documents: Legal frameworks and international guidelines

Document	Origin	Geography (institutional ties, if any)	References	Referred in the article as
International Ethical Guidelines for Health-related Research Involving Humans	The Council for International Organizations of Medical Sciences	International (WHO and UNESCO)	Council for International Organizations of Medical Sciences (2016)	*CIOMS*
General Data Protection Regulation	European Union	Regional/Supranational	European Parliament and Council of the European Union (2016)	*GDPR*
Health Insurance Portability and Accountability Act	USA	National	US Department of Health and Human Services (2012)	*HIPAA*
Common Minimum Technical Standards and Protocols for Biobanks Dedicated to Cancer Research	The International Agency for Research on Cancer	International (WHO)	Mendy et al. (2017)	*IARC*
Best Practices: Recommendations for Repositories	International Society for Biological and Environmental Repositories	International	Astrin et al. (2018)	*ISBER*
ISO 20387:2018 General Requirements for Biobanking	International Organization for Standardization	International	ISO (2018)	*ISO*
Guidelines on Human Biobanks and Genetic Research Databases	The Organization for Economic Co-operation and Development	International	OECD (2009)	*OECD*
Declaration of Taipei on Ethical Considerations regarding Health Databases and Biobanks	The World Medical Association	International	World Medical Association (2016)	*WMA*

Considering that biomedical research practice involves “ethics work” which is central for the flow of data (Hoeyer et al. 2016) as well as further “data work” in making the data ‘actionable’ in healthcare setting (Fiske et al. 2019), the approach taken here does not focus on how these documents are used or related to in actual research practice, but instead the selected documents were analyzed as potential resources at researchers’ and research infrastructures’ disposal. The interdisciplinary character of the co-authoring team and their involvement in the European biobanking research infrastructure (BBMRI-ERIC) have been useful in this respect. To illustrate, the co-authoring team, comprised of scholars in law, bioethics, social sciences, and biomedical sciences, have been involved with biobankers and life sciences researchers in different capacities, e.g., as researchers, collaborators in international projects, or experts on ethical, legal, societal issues providing guidance on a wide range of issues, including informed consent for research using biological data and samples, material and data transfer agreements, incidental findings, public perception and trust. Hence, utilizing the reciprocal relationship between expert and expertise from the analytical perspective of sociology of expectations allows to gain insights on institutionalized knowledge, thus making better sense of the HeLa 2.0 and how identifiability was dealt with within this episode.

## Findings

Findings based on the analytical framing of sociology of expectation and the discursive analytic approach are summarized through the case of HeLa and the risk of genomic identifiability in analyzed documents.

### HeLa 2.0 as a case: from uncritical welcome to a matter of critique

The HeLa cell line has been embroiled in controversy since its origin in 1951. The specific episode that this paper builds upon began in 2013 when a research group at the European Molecular Biology Laboratory (EMBL) published an article (Landry et al. 2013) in which the authors provided the HeLa genome as supplementary data in line with the prevailing efforts toward open access/data. EMBL publication was initially a celebrated development for the scientific community, providing a high-quality genome sequence of the cell line. One week after publication’s appearance in the journal *G3: Genes, Genomes, Genetics*, an uncritical news article in *Nature* with the title “Most popular human cell in science gets sequenced” announced the research as a success, stressing the potential uses of the sequence for science (Callaway 2013c). A celebratory press release had also appeared on the EMBL website, where several questions were listed, including “Can we infer anything about Henrietta Lacks or her descendants from this sequencing?”, the answer to which began with, “No, we cannot infer anything about Henrietta Lacks’ genome, or of her descendants, from the data generated in this study.”^1^ Soon after, however, it became clear that not everyone shared this opinion.

On March 19, 2013, an announcement from EMBL appeared on the *G3* journal’s website, which stated, “relatives of Henrietta Lacks expressed concerns about the ways in which genetic data from HeLa cells may affect their privacy” and the authors “are currently withholding these data out of respect for the Lacks family”.^2^ It was not only the Lacks family, but a larger community of scientists, journalists, and individuals who had concerns. One day before the data were taken down, Jonathan Eisen, a biology professor at the University of California-Davis, tweeted “A bit stunned that the people publishing #HELA genome appear to not have gotten consent from the family” bringing the matter of consent to the fore (Eisen 2013b). On March 24, Skloot (2013) wrote in *The New York Times*: “The publication of the HeLa genome without consent isn’t an example of a few researchers making a mistake. The whole system allowed it. Everyone involved followed standard practices. They presented their research at conferences and in a peer-reviewed journal. No one raised questions about consent.”

#### The ‘other’ parties becoming involved in the institutional negotiations

Following the initial criticism of the lack of consent of the family, the Genetics Society of America—the owner of the G3 journal—announced on March 26 that the article would remain online; however, the data would be unavailable until an agreement between “the researchers and other parties” was reached.^3^ Although this has not been noted in the announcement, the other parties included the Lacks family representatives, and the negotiations mainly took place under the roof of the NIH with Francis Collins as the director of the NIH attending the meetings (Callaway 2013a). Following the three meetings with the family at the Johns Hopkins University campus, it was agreed that the data would be available to the researchers as announced on August 7 in *Nature* (Callaway 2013d) and *the New York Times* (Zimmer 2013). However, access would be possible only after the submission of a proposal to an NIH Committee, which also included other Lacks family members.

NIH’s interest in a deal with the family was possibly manifold as it had provided funding for a similar project in 2011, having led to a paper submitted to *Nature* in 2012 and was already accepted at the time the controversy erupted (Callaway 2013a). As Callaway notes, the “paper’s reviewers did not raise privacy concerns before recommending it for publication; nor did *Nature*” (Callaway 2013a, p. 133). This second HeLa genome paper (Adey et al. 2013) by a genomics group at the University of Washington was eventually published in *Nature* in the same issue in which the deal had been announced. Currently, access to the data is possible only through NIH’s Data Depository (dbGaP) following an application and approval by a board.^4^ As of September 2022, there have been 88 authorized requests for the deposited data from academic and non-academic researchers around the globe (dbGaP, n.d.). While announcing the deal, the directorship of the NIH highlighted the uniqueness of the situation and stressed that this should not set a precedent for future: “[W]e are responding to an extraordinary situation here, not setting a precedent for research with previously stored, de-identified specimens” (Hudson and Collins 2013, p. 142).

#### Negotiating (the nature of) the data in public: a spectrum

The controversy seems to have been resolved in a relatively short time. During this time, different actors had been communicating with each other and the public in various ways. While Rebecca Skloot supported the family during the negotiations with the NIH (Hudson and Collins 2013, p. 142), the EMBL research group received “a bench-side ethics consult and […] advice” from professionals (Greely and Cho 2013, p. 849). However, during the negotiations between NIH and the family from March until August 2013, the discussion of the controversy did not cease in the public arena. A broad spectrum of views on social media, scientists’ blogs and newspapers reveals how certain points had to be negotiated in the public sphere, especially regarding the nature of the genomic data—its power to reveal (e.g., health risks for relatives) and its availability (e.g., in databases or at the disposal of research groups).

A salient point during the debate was how scientists disagreed whether the data could reveal anything about the family members. Initially, EMBL research group defended that the data would not reveal anything about Henrietta Lacks’ or her descendants’ genomes, providing two reasons in EMBL’s original press release: the genome of an individual obtained from a tumor would differ from the individual’s genome in healthy somatic cells, and further changes in the HeLa genome emerged over time as the cell line continued to grow.^1^ Yaniv Erlich, already known for showing how genomic data from public sequencing projects could allow identification of the participants through comparisons with recreational genomics databases (see: Gymrek et al. 2013), countered this statement, tweeting “Nice lie EMBL!” (Erlich 2013). EMBL finally modified its press release, removing the sentence “No, we cannot infer anything about Henrietta Lacks’ genome, or of her descendants, from the data generated in this study” and by adding a sentence that reads “The data generated in this study therefore does not change the fact that it is possible to make predictions about Mrs. Lacks’ genome, or those of her descendants”.^1^ This change was striking, especially considering that the necessity for consent of the family could only be based on the argument that the published data revealed information of Lacks family members other than Henrietta Lacks. Looking back at this episode, David Lacks (Henrietta Lacks’ grandson) is quoted in 2017: “We could leave it out there as is, for the whole world to see, but the issue with that is when you sequence Henrietta Lacks’ genome, you also include family traits of our genome as well […] We don’t know what would be known 20 years from now with that sequence just being out there for anybody to use and how that would have an effect on us” (Arnst 2017).

The second central argument was that the existence of large amounts of genomic data from HeLa already available online makes the 2013 releases of the HeLa genome irrelevant. According to the argument, the widespread availability of HeLa cells and the capacity of laboratories to sequence its genome would make it futile to block the online availability of HeLa genome data. In fact, it was argued that many research groups around the world already had such sequences at their disposal and snippets existed in databases such as ENCODE (National Human Genome Research Institute 2021). The authors were quoted in *Nature* at the height of the controversy: “If we take our data off it doesn’t change anything […] There are more data already out than what we generated in our study” (Callaway 2013b). Others brought publicly available data together to show that even before the Landry et al. (2013) paper, the genomic data available on HeLa was immense and the new release was not a major change.^5^ Problematizing the previous data in ENCODE, a central tool for research, scholars like Jonathan Eisen asked why Francis Collins (NIH) and the creators of ENCODE did not think about the identity of Henrietta Lacks being known when the platform published genomic data from the HeLa cell line (Eisen 2013a). Yaniv Erlich claims, some feared the discussion would be detrimental to science if all such databases could be closed down due to privacy concerns and consequently people would be more inclined not to participate in genomic biobanks (Hayden 2013, p. 174).

#### The aftermath

Caulfield and McGuire (2013) suggest that HeLa 2.0 raises two questions that go unanswered, especially from a legal perspective. On the one hand, the legal framework is unclear regarding ownership and control of biological samples such as HeLa; on the other hand, even if there were to be a legal framework, where relatives could be asked for consent, it is also unclear how far down family lines the consent should be sought since genomic data is shared across relatives to varying degrees (Caulfield and McGuire 2013, p. 1206). It also turns what used to be donor’s protection or anonymity into a family issue (Kuehn 2013), while genetic privacy is problematized as a continuum rather than a binary state.^6^ Some of these aspects were previously foreseen in bioethics reports, particularly blood relatives in genome sequencing “who most likely did not consent to the sequencing procedure” extending concerns over privacy beyond the individual who consents to sequencing (Gutmann et al. 2012, p. 2).

At the height of the controversy, David Kroll is quoted in *The Guardian* considering the case “a blessing in disguise” that may lead to positive change (Harris 2013); looking back at this episode, it has yielded suggestions for responsible research practices and possibly greater awareness. For instance, Szego and others suggested five questions for researchers and journal editors, which direct the researchers to “ethics review” or “research ethics consultation services” (2013, pp. 1210–1211). More importantly, however, HeLa and other recent controversies were raised in the debates regarding the recent Common Rule change in the US (Kaiser 2016). In an Op-Ed in *The New York Times*, Skloot (2015) called on people to raise their opinion in the proposed Common Rule change by stating the questions: “Should scientists have to ask permission to use all leftover clinical samples? Would you say yes? Is broad general consent enough, or do you want options for more control? Why? Should this apply to both tissues and genetic information, anonymous or not? And what if this slowed scientific progress?” It is difficult to say the momentum of the HeLa 2.0 was converted into substantial change in the practices, but it has initiated a discussion that we want to rekindle by highlighting how this is not an incident of the past but an opportunity to think about the future.

### Guidelines and legal framework: (not) constructing genomic identifiability as a risk?

A brief contextualization of the current possibilities and developments with regard to genomic data, their identifiability, and intensified utilization is necessary before presenting further findings. Genomic data is highly distinguishable, considering that with only 30 SNPs (i.e., single nucleotide polymorphisms: a type of common variation on the genome), the individual can be identified (Dankar et al. 2018). With the evolution and expansion of -omics technologies, the sources of genomic data are increasing, genetic/genomic databases are expanding through research collaborations, and vast amounts of masked or aggregated genetic data are being stored, analyzed, and provided to researchers (Knoppers et al. 2007; Karczewski et al. 2020; WHO 2002). Outside the healthcare/research systems, large pools of genetic data are generated by private companies offering various recreational, direct-to-consumer (DTC) genomics services (Thiebes et al. 2020). The users of these services can voluntarily introduce their genotypic and phenotypic data to other online platforms (such as GEDmatch), some of which are granting open access to their data for further recreational or forensic purposes (Erlich et al. 2018; Drabiak 2017), others enable research participation by individuals, including sharing of personal genomic and health-related information (Borry et al. 2018).

In the context of an increasingly datafied genomics landscape and growing potential of identifiability as the point of departure, we have analyzed the documents listed in Table 1. While documents are often studied by social scientists through their capacity to represent the world, they are also constitutive in character in that they are embedded in knowledges and objects of different types, but also in subjectivities, practices, rules, and organizational structures (Shankar et al. 2017; Hull 2012). As the broad array of literature shows, risk is a complex concept and is often discussed in STS through expertise, uncertainty, and ignorance, but also democracy, representation, and participation, especially in relation to biomedicine (Hilgartner et al. 2015; Irwin and Wynne 1996). In this regard, a shortcoming of the approach taken in this paper is that it is focused and situated within a Western, mainly European, framework, and as it is not ethnographically oriented, it does not allow following how the documents are involved in the practices or how these documents are produced (e.g., the legal deliberations or political discussions during legislative practices). Rather, in line with the aforementioned analytical framework, our focus has been on revealing how the risk of genomic identifiability has (not) been constructed and what kind of risk assessment, communication, and mitigation practices are discussed in the selected documents.

Generally, biobanking guidelines broadly describe the technical requirements for collection and processing of biological material as well as different types of data. We found that the documents analyzed do not directly define genomic identifiability or thoroughly discuss the risk of genomic identifiability. For example, both the *IARC* and *ISBER* guidelines address the problem of identifying patients whose genomic data is generated and shared within research projects; however, this takes the form of general advice to follow the local legislation, regulation, and guidance (*IARC*, Section 3.1.3 Data Protection, Confidentiality, and Privacy) and the scope of the consent given by the research subjects, as well as to have both an appropriate data management system to protect against misuse of data and a policy that outlines the terms and conditions of its use (*ISBER*, L.2.6 Specimens to be Used for Genetic Analyses; L.2.7 Sharing and Distribution of Specimens). Although the issue of re-identification of the individual based on genomic data has been mentioned, for instance in *IARC* (in Section 3.1.4), it is discussed merely as an aspect the researcher has to be aware of, both when generating this type of data and safeguarding against their improper access/use, similar to the discussion of personal data protection.

Identifiability has been raised as a concern on multiple occasions, where it is acknowledged that “Traditional de-identification techniques have notable limitations, and definitions based on a simple concept of ‘identifiability’ lack sufficient precision to be used as a standard” (*CIOMS*, p. 84). Identifiability is often part of the discussion of anonymization and safeguards toward the protection of privacy and confidentiality. Broadly, anonymization of or coding/encrypting data are constructed in multiple ways. On the one hand, anonymization is provided as a solution to “prevent subject re-identification” (*OECD*, p. 45) and to “protect the confidentiality of the information linked to the material” (*CIOMS*, p. 41). On the other hand, such practices are also constructed as part of broader governance mechanisms, supported by other structures, such as data enclaves or honest broker systems (*OECD*, p. 36). While coding/encryption is not problematized, anonymization is discussed as a block to certain processes, by potentially preventing the participant’s own access to their samples/data (*OECD*, p. 37) or return of results/(un)solicited findings to the participant (*CIOMS*, p. 51), and therefore creating new ethical issues. Finally, anonymization is also constructed as not completely feasible, and as “becoming increasingly illusory as the possibility of cross-matching large datasets improves” and therefore arguing that “the more difficult it becomes to anonymize data, the more important it will be to retain the ability to remove personal data from a dataset” (*CIOMS*, p. 44).

Infrastructures are “material forms that allow for the possibility of exchange over space” (Larkin 2013, p. 327) and the HeLa case relates not only to disruptive technologies but also to the ways in which the exchange becomes possible. In this respect, we focused on issues relevant for genomic identifiability as a risk that have been sidelined or completely ignored in the guidelines. It has been noted that the biobank should consider that “genetic data held by it might allow the identification of participants, either alone or in combination with other available data and reference samples” and “establish a clearly articulated policy of whether certain data or combinations of data will not be made available and for which reasons” (*OECD*, p. 13). Moreover, the risk of cross-matching large datasets becomes a further concern as mentioned above (*CIOMS*, p. 44). Aforementioned emerging data infrastructures, such as GEDmatch or OpenHumans.org, may also lead to cross-matching; however, no direct reference to such non-academic infrastructures or genomic data in academic open access databases is made in the guidelines.

Considering the spectrum of the processes to prevent identifiability as discussed above, we note the following aspects that are not systematically considered, even if mentioned: immortal cell lines that may be generated and made widely available (*OECD*, p. 42; *CIOMS*, p. 42), death of the participant and posthumous generation of genomic data from samples (*OECD*, p. 33), genomic data generated from samples by third parties following a Material Transfer Agreement (*OECD*, pp. 39–40). The limits of confidentiality and need to mitigate risks are acknowledged and such concerns are often addressed by basic suggestions such as obligation of the researcher not to attempt to re-identify individuals (*OECD*, p. 14) or stating that “The existence of data and information already online does not relieve the researcher from the obligation to respect privacy and mitigate risks that could result from combining data from multiple sources and their subsequent use and publication” (*CIOMS*, p. 84). Lastly, in line with the main purpose of the document, the only ISO document analyzed (*ISO 20387*) does not provide specific solutions or rules to avoid the identification of the participant based on genomic data, but it specifies various aspects related to the quality management system, including appropriate validation of technological solutions used in biobanks, as well as monitoring of adverse effects that may occur during the operation of such a unit. In this regard, genomic identifiability as a risk might be too specific to be discussed within this document.

We have also considered legal texts (*HIPAA* and *GDPR*^7^) which are crucial in informing other documents such as the guidelines that we have analyzed. These documents set limitations and shape practices. For instance, the *GDPR* provides a dynamic legal framework for personal data protection based on a proactive and systematic privacy breach risk assessment. In a sense, it becomes a “living document” through working parties, continuous public consultations, and instruments such as codes of conduct that adapt the *GDPR* to sociotechnological realities of different communities and contexts.

The *GDPR* discusses numerous actors, such as data controllers and data processors, who, regardless of the function, are required to undertake by design and by default, all necessary technical and organizational measures and safeguards to protect personal data from individuals located within the EU. The *GDPR* defines personal data as any information allowing direct/indirect identification of the individual (data subject), thus including pseudonymised data (coded/encrypted with a key linking to data subject’s identity) and excluding anonymous and anonymized data. Genetic data is legally defined for the first time and inscribed in the special category of personal data due to the sensitivity of their processing regarding risks for data subject’s rights and freedoms. A structure organized under the previous Data Protection Directive (replaced by the *GDPR*), named “Article 29 Working Party” (WP29) (replaced by the European Data Protection Board) issued an opinion stating that data should be considered anonymous provided that their anonymization is ‘irreversible’ (Article 29 Data Protection Working Party 2014a).

Interestingly, Recital 26—contextualizing information for the relevant article in the *GDPR*—adopts a risk assessment approach distinguishing whether data should be considered anonymous or not based on whether re-identification of the individual is “reasonably” likely to happen or not (Finck and Pallas 2020). This might prove rather problematic in practice, as the interpretation of “reasonably” varies both temporally and spatially, across national jurisdictions and practices and it depends highly on context. Personal data considered non-identifiable at the time of its collection may be rendered identifiable at a later stage, since genetic datasets, for example, allow more connections to be drawn making identification possible (Harbord 2019). As a consequence, the risk of matching anonymized genetic data with data from other datasets, in the ongoing evolving research environment, is a dynamic process, progressively increasing the possibility of data subject re-identification (Shabani and Borry 2018). Re-identification risk is also increased through the sharing of genetic data on third-party platforms, through which individuals may be re-identified from genetic data of their distant relatives, even though they have never provided their own DNA to a database (Erlich et al. 2018) with recent examples of forensic use of genomic data of relatives and the third-party platforms at a third-country for solving longstanding criminal cases (AFP Sweden 2020). While these examples allow the discussion of what anonymization means, they also relate to the datasets themselves. In order to minimize the risks of re-identification, several factors such as the characteristics of datasets and the context in which the processing occurs should also be considered when processing genetic data (Data Protection Commission of Ireland 2019). Considering that more advanced data processing techniques will be developed and that additional data sets will be released into the public domain, allowing for enhanced cross-comparison between vast amounts of genetic datasets (Harbord 2019), an individual’s genomic data cannot be considered alone.

Despite the observation that the stronger the level of de-identification, the lower the privacy breach risk and vice versa (Hintze 2018), de-identification is not conceptualized in the same way on both sides of the Atlantic. In fact, as Voss and Houser (2019) note, “Comparing personal data as the term is used in the EU to personally identifiable information as the term is used in the United States, is like comparing apples to oranges.” More specifically, when it comes to the de-identification of health data in the US, this is perceived as the process of removing 18 specific identifiers, considered to be personally identifiable information, such as the data subject’s name, telephone number or address, removal of which then allows, according to the *HIPAA* [§ 164.514(b)], the data to be freely shared without any other restrictions (US Department of Health and Human Services 2012). In contrast, the European legislator suggested pseudonymisation as merely one of the safeguards and technical measures which controllers and processors may use in order to ensure the data subjects’ data protection, thus abstaining from proposing specific de-identifiers (which could actually decrease the level of protection afforded by the technique). While the *GDPR* considers pseudonymised data as protected personal data, health data in the US context can be freely transferred after the *HIPAA* de-identification procedure takes place. This underlines the necessity for a broader understanding of the full range of data processing activities which fall under the term “de-identification.”

Application of safeguards such as pseudonymization or stripping off specific identifiers is not a panacea or a “highway” to full data protection. In fact, as ISO/TS 25237:2008(E) states, “[a]nonymization is another subcategory of de-identification” where de-identification covers a wide spectrum of dynamic data processing activities which may or may not end in the complete anonymization of datasets (Garfinkel 2015). In the *GDPR*, data controllers are in certain cases obliged to practice a careful and detailed pre-assessment ensuring that the principles of data protection by design and by default have been met, notably before adopting the appropriate technical and organizational measures and safeguards. Such pre-assessment is called a “data protection impact assessment” (DPIA). We will briefly consider this practice for better contextualization.

Prior to genetic data processing in the EU, a DPIA is often mandatory since such processing meets the criteria characterizing processing likely to involve or to result in a high risk regarding privacy rights (Article 29 Data Protection Working Party 2017). A DPIA aims to consider proactively, prior to the start of the processing and based on its specific scope and context, the actual impacts on data subjects’ fundamental rights and freedoms, with plurality of solutions in terms of technical and organizational measures that permit adequate protection of data subjects’ interests while keeping the data functionality and usability by the data controller (Article 29 Data Protection Working Party 2017). Considering the data lifecycle approach, it is a method to facilitate proper data governance which necessitates documentation and regular updates. Regardless of “the nature, scope, context, purposes of the processing and the risks for data subjects,” the controllers are expected to be accountable for compliance while respecting the rights of the data subject even in the case of risks related to the data processing, such as “right of access, rectification, erasure, objection, transparency, right to be forgotten, right to data portability” (Article 29 Data Protection Working Party 2014b). In this regard, for the *GDPR*, rather than a narrow definition of harm, the focus is on the risk; however, the expertise necessary for the data controller to make an informed judgment regarding genomic data is not straightforward, as the HeLa 2.0 shows.

The documents analyzed are important instruments in shaping biomedical research practices. The patchiness of how genomic identifiability is constructed in such documents highlights that it is an evolving concept that necessitates a practice-focused analysis. As a well-known controversy, the HeLa cell line and the discussions around the HeLa genome serve as a case that complements our analysis of the selected documents regarding how genomic identifiability is constructed as a risk and opens up the very concept of identifiability and what it means in a datafied world.

## Discussion

Studying how genomic identifiability is constructed as a risk necessitates understanding the sociotechnological changes, especially the evolution of the research and data environment. Our analysis of documents in this article mainly relied on a pre-genomic imaginary of the world. Unlike this pre-genomic world, the world that we inhabit is moving toward a postgenomic one (Reardon 2017; Stevens and Richardson 2015), in which genomic data is being generated at an incredible rate due to the developments in sequencing and genotyping technologies and infrastructures to analyze and link various data, as well as by applying advances in artificial intelligence and deep learning (Koumakis 2020). Along with such changes and the expansion of the spectrum of generated genome sequences—from human groups with certain health conditions to epigenomes of individual cell lines, from microbiome of the human gut to the genomes of coronavirus strains that originate in different parts of the globe—the understandings of genes and genomes change (Fox Keller 2014, 2015; Dupré, 2015). The postgenomic world is marked by an increased size and speed, but also by the involvement of the non-academic, often venture-capital supported entrepreneurial efforts of value-making (Waldby and Mitchell 2006), such as DTC genomics companies, crowdsourced projects or citizen science as well as the emergence of new identities for the previous roles of the ‘donor’ or the ‘research subject,’ such as the consumer, the participant, and the data subject. In this world, biobanks—public or private, population-based or disease-oriented, health or ancestry—are racing to collect, analyze, and connect genomic and other personal data.

### HeLa 2.0: a specific case vs. a systematic data issue

The transition from the pre-genomic to the postgenomic world has not been a sudden change, despite the existence of landmark moments such as the announcement of the completion of the Human Genome Project or the reaching of the “psychological price threshold” of USD 1000 for whole genome sequencing cost (Pinxten and Howard 2014, p. 270). We have followed a crucial episode in the postgenomic world, the HeLa 2.0, and tried to make sense of what this controversy means for ‘genomic identifiability’ in light of the documents that may be guiding researchers’ practices around biological samples and genomic data. While we believe in the importance and contribution of case studies, our analysis has led us to conclude that despite some of the actors’ efforts to construct the HeLa 2.0 as a specific case, or even an idiosyncrasy due to the past of the cell line and the lack of informed consent in the beginning, it is part of a phenomenon that allows understanding genomic identifiability as a systematic data issue, and we will show this by situating HeLa 2.0 within broader developments.

In the pre-genomic world, the limiting factor for widescale sharing of large amounts of genetic data was not only the relatively small amount of data at that time, but even for the existing data, the expertise to understand and use the data, available methods of data analysis and possibilities of sharing were largely different than today’s genomic data environment. Currently, large biobank collections, including some public and private genomic data collections, aim for continuous and global use of the samples and data for research purposes in accordance with the rules and guidelines applicable in their respective countries. In comparison to smaller-scale research group repositories of the past, such infrastructures allow easier access for external researchers as long as the standardized approval processes are followed. However, third-party platforms, such as GEDmatch, are also indirectly housing genomic data that has been generated not by scientists, but through DTC genomics services (Erlich et al. 2018). In such a system, neither the use nor the production of the data is similar to the past research-focused data generation. Furthermore, DTC genomics services and third-party platforms often allow contact between individuals who upload their genomic data, removing barriers against identifiability which, in biobanks, are often in place by design and are controlled. Thus, individuals may lose their genomic anonymity due to their biological relatives’ data practices, even if they do not contribute any of their genomic data. In other words, in the postgenomic world, an individual’s sharing of their genome in an identifiable manner might automatically render any supposedly unidentifiable genomic data of biological relatives identifiable, without their consent. In this regard, the genomic identifiability risks for Henrietta Lacks’ biological relatives are similar to the situation for each individual whose close biological relatives link their genomic data to their identity in public databases.

NIH’s efforts to maintain that the HeLa 2.0 is “an extraordinary situation” to prevent “setting a precedent for research with previously stored, de-identified specimens” (Hudson and Collins 2013, p. 142) is reflective of HeLa exceptionalism that marks this debate. Compartmentalization of the controversy allows continuation of the current practices, while the specific case is constructed as a consequence of past research practices that are supposedly preventable with current standards. Thus, Hudson and Collins’ concerns regarding legacy samples overshadow broader discussions that might have otherwise taken place on the peculiarities of genomic data and data environment in a postgenomic world.

Despite the intentions to paint HeLa as exceptional, discussions or even practices that run counter to genomic privacy as a concept are increasingly becoming part of the research arena. There are efforts, such as the Personal Genome Project Global Network, where participants are asked to consent to public disclosure of their genomic data, where it is explicitly stated that the projects do not promise anonymity or confidentiality (Open Humans Foundation, n.d.). For instance, the participant has to consent even to the risk of use of the individual’s genome sequence by others to “make synthetic DNA and plant it at a crime scene, or otherwise use it to falsely identify or implicate you and/or your family” as well as acknowledging that “the PGP cannot predict all of the risks, or the severity of the risks, that the public availability of this information may pose to you and your relatives” (Church 2017, p. 15). Furthermore, the use of genomic ancestry data, such as GEDmatch platform, to solve criminal cases, e.g., the Golden State Killer, further entrenches identifiability through biological relatives’ genome as a standard approach in such investigations to the extent that a field of its own has emerged: forensic genealogy (Phillips 2018). Consequently, rather than guidelines (e.g., *CIOMS*) or legal frameworks (e.g., *GDPR*) setting standards on genomic privacy, the emerging open access practices around genomic data suggest that ‘identifiability’ is neither a specificity of the HeLa 2.0 nor a rare risk anymore, but part of postgenomic data practices.

### Post-identifiability

The considerations above open up a space to discuss meanings of anonymity and identifiability in the context of more actors and institutions involved in the data environment along with an increasing datafication of more areas of life. We, as the co-authors, ask how to think differently about understandings of anonymity and identifiability, more in terms of post-identifiability? In her book *The Postgenomic Condition*, Reardon (2017) argues for a re-thinking of the condition in which we find ourselves, owing to the literature from Arendt and Lyotard and the observation that computational power and genomic data are united in infrastructures that span the globe. In her conceptualization, the question is not only which matters of concern will be central to our daily lives, but also “whose cares and concerns should matter” within the triangle of knowledge, ethics and justice, with conflicting realities such as the use of genomics to prove that there is only one human race as well as its use to claim racial differences (Reardon 2017; de la Bellacasa 2010; Latour and Weibel 2005). The postgenomic condition signifies a shift in producing knowledge about life and making meaning of it as well as meaning of the concepts of ethics and justice in a datafied genomic world. In such a world, identifiability is constantly challenged as a concept that does not match the realities of the day. Thus, with this article, we propose a re-thinking of identifiability in terms of *post-identifiability* as a sociotechnological situation that we find ourselves in, with a multiplicity of actors, sites, practices and futures involved.

We see post-identifiability reflected in the idea of data protection being proactive and collective vs. the conceptualizations of data from the past as individual personal data (unique data subject, etc.), which show how past assumptions and ideas about future possibilities come together in present debates as illustrated above. Evolved understandings of genetic identifiability have set the path to locate the issue in the anonymity discourse, or “the fiction of anonymity” (Émon 2017, p. 2), but the sociotechnological context, i.e., the data environment, has changed. Anonymity is used to manage the disparity between on the one side legally and societally developed norms and rules and on the other technological possibilities in a highly datafied world. The “past futures” leading to present data safety and security, anonymization, and consent approaches are also generating fictions in (future) presents of increased needs of access to and sharing of (big) genomic data resources for research purposes. For instance, anonymization is meant to protect the individual, but in some medical circumstances, re-identification could be desirable. The “past” of the anonymity discourse also points to a change in the risk profile of data use (Mikkelsen et al. 2019), which primarily focused on the individual, but postgenomic big data also affects relatives, as the HeLa case shows, and groups of individuals, e.g., ethnicities (Mittelstadt and Floridi 2016; Kasperbauer et al. 2018). In this situation, different understandings (e.g., public and private) and uses of data co-exist, leading to the suggestion that “Individual control is an inadequate measure for ensuring privacy interests” (Hoeyer 2020, p. 6) as well as the need for a “networked privacy” (Boyd 2012).

Anonymity is one approach to manage current challenges regarding identifiability; consent is another (Mostert et al. 2016). The above argument could also apply, as informed consent of the past does not match the post-identifiability reality. In this regard, even if Henrietta Lacks had provided her consent for research on the tumor biopsy sample, the realities of the day would have allowed neither the participant nor the scientists to foresee today’s debates of identifiability. Current consenting practices are supposed to protect the individual and meet institutional structures of risk mitigation, but they are also expected to allow potential and unknown future (big) data uses. The challenges foreshadow the question whether broad consent is ‘deep’ enough (Mikkelsen et al. 2019), or the functionality of standard safeguards: for example, a data access committee’s decision to allow data access for a study on sexual orientation on the basis of a health-related research (Goisauf et al. 2020). We can also see that consent and anonymity are conflated in a way that consent may exclude guarantees of anonymization of genomic data (Church 2017).

### Opening up post-identifiability: identifying the current problems

New technologies, such as large-scale genomic data infrastructures, bring with them uncertainties, and it is possible that the understandings and expectations of the risks of new technologies are often tied to those of current and past technologies. Defining ‘genomic identifiability’ as a risk of an individual confines the issue within boundaries of a known, but a past world of data with its distinct practices, infrastructures and scope as well as distinct ways of ‘taming’ the risks. However, the continuously expanding multiplicity of actors, institutions, regulations and practices turns the seemingly individualized risk into a systematic issue in a datafied postgenomic world, where genomic data gain relevance not in its alienated form, but in practices of being embedded in comparisons with other data.

Despite the uncertainties and risks of new technologies, there are often systems in place to counter these: in the case of biomedical research infrastructures, for instance, internal measures, international guidelines, community standards that ensure safe handling of the data in the databases (Holub et al. 2016), as well as typologies of risks (Akyüz et al. 2021). In this regard, following the latest security standards could lower the identifiability risk for participants significantly; however, these relate to the data stored at the institution where these practices are enacted. At the same time, these have their own limits: for instance, standard operating procedures, DPIAs, ethics self-assessments and risk assessments are useful as long as they are up-to-date or performed regularly. Such standards and standardized practices produce realities of their own, but what is applicable depends on circumstances and the special context (Timmermans and Epstein 2010). The standards and standardized practices change over time.

The consequences of intensification of the interconnectedness, expansion of the data world, management of data not at a single institution but globally and not merely today, but in the futures that are being made today are something to consider in further investigations. In this regard, as part of post-identifiability, issues and concerns emerge that may not have easy solutions and are often political as much as they are technical. Three axes are central to understand post-identifiability: kinship, temporality, and openness.

#### Identifying the individual’s data: kinship on a spectrum

As HeLa 2.0 and the broader context of the current genomic data environment suggest, collecting/analyzing genomic data may affect not only the person who has provided the biological sample and consented to genomic analysis, but also their relatives raising further issues beyond health becoming a “‘family’ matter” in the genomic age (Novas and Rose 2000, p. 490). This raises several normative and practical questions. In which cases and how should consent from family members be obtained? What are the researcher's duties toward the relatives of research participants? How can a relative's right to withdraw be implemented? How does sharing genomic data together with other medical and pedigree data increase the identifiability risks? How should the additional risks when sharing genome data of several family members be managed considering that “the risk of re-identification of data contributors increases when sharing the clinical and genomic data of patients and family members” (Takashima et al. 2018, p. 5)?

To answer these normative and practical questions, some authors have started with defining the role of a relative who is not directly participating in the research. This may take the form of establishing a difference between primary and secondary research subjects, i.e., “[t]he research subject interacting with investigators and answering questions” and “relatives about whom identifiable private information is collected,” respectively (Resnik and Sharp 2006, p. 2). It may also rely on the use of terms: “active participant (proband)” and “passive participant” (Worrall et al. 2001). In response to a controversy at Virginia Commonwealth University, in which the father of a consenting twin study participant expressed concerns about threats to his privacy and lack of his informed consent to this type of research, the NIH ruled that the responsible ethics committee “should have considered whether family members were human subjects of this research by virtue of their relationship to the respondent and the nature of the family information obtained from the respondent” (Botkin 2001, p. 207). However, so far there still is a lack of harmonized international practice regarding the status, consent/waiver of consent, and protection of relatives of research participants. Some authors suggest general solutions like developing “improved informed consent models” or asking potential research participants “to have a discussion with specific family members before proceeding” (Cassa et al. 2008). Although the issue has traditionally been considered from the perspective of rare disease research, there is ongoing discussion into involvement of the family members to the extent of hypothetically thinking of “genetic family as a patient” (Knoppers and Kekesi-Lafrance 2020).

#### Temporality: meaning of consent in time and expanding data-space

Storage of biological samples and genomic data and their long-term use reveal myriad issues, especially regarding the validity of initial consent to future unforeseen uses of the data; this is often considered from an ethical and legal perspective. In this respect, legal mechanisms exist for facilitating broadened individual consent regarding further genetic data processing in research, whereas renewal of consent as a practice is part of the ethics debates. Along with the postgenomic transition and potential unforeseeable uses of samples and data, uncertainties may emerge over time about the expressed wishes of the participant through changes in the individual’s situation (e.g., acquisition of legal capacity at majority age), but also in the research purposes (e.g., consent to store samples in a biobank does not necessarily mean consent to derivate cell lines and commercial uses), technological change (e.g., consent to targeted testing does not necessarily mean consent to whole genome sequencing) or other significant changes (e.g., institutional changes due to a biobank closure or custodianship transfer). While legal and ethical concerns are part of the picture, concepts such as ‘dynamic consent’ (Kaye et al. 2015) highlight how practices of and technological framework for consent get transformed, yielding a new temporality, in which research participants can give or specify consent to certain uses along the way, toward a new paradigm in health research based on improved communication and participant engagement (Teare et al. 2021). Considering the collection and use of data and samples as part of a life cycle, genomic post-identifiability may foster further discussions on the temporality of consent.

Unlike samples that are often limited in quantity and cannot be replaced unless they are already transformed (e.g., immortal cell lines), genomic data is amenable to continuous use, even after the death of the participant. Both from an ethical and legal perspective, the post-mortem use of genomic data and informational privacy are complex issues; some regulations like the GDPR do not apply after data subject’s death and states seldom introduce legal provisions in this regard (Chassang 2021). Acknowledging the difficulty, and, probably, impossibility of anonymization of genomic data and the potential of the genomic data processing to reveal information about biological relatives, post-mortem privacy policies could at least serve to inform individuals about the fate of their genomic data and the way in which it will be managed after death and avoid certain complexities when broad uses are envisaged as in the case of HeLa. As part of post-identifiability, the questions emerge regarding potential rights for the living genomic data subject to prepare their ‘informational legacy’ as well as for known biological relatives of the data subject, for instance, where significant and actionable health information result from research activities performed with samples or data of a deceased relative. While post-identifiability brings new questions and concepts to the data landscape, the meaning of consent is being renegotiated, from dynamic consent to familial consent, opening up further questions into what entails ‘biorights’ (Caulfield and Murdoch 2017) as much as what comprises ‘genomic commons’ (Contreras and Knoppers 2018).

#### Opening science, engaging citizens: from protecting privacy to data altruism

The concerns regarding the risk of genomic identifiability are part of the postgenomic landscape, but this is in multiple ways tied to our understanding of science and technology in the twenty-first century and the importance of access to various types of data. Making data FAIR (Wilkinson et al. 2016) is expected to contribute to science and technology; however, when the data in question are human genomic data, the principles do not simply correspond to practices for protecting privacy and limiting risk of identifiability. In other words, the aim to open science by opening data raises the concerns of potentially legitimizing practices that would be considered blanket consent due to broadness and the unpredictability of the potential uses of genomic data. At the same time, the expansion of DTC genomics services and the spread of data generated by these to third-party platforms along with the global mobility of genomic data through research infrastructures make evident the distinct ways of dealing with data across national borders and academic/non-academic research alike. In a postgenomic world, the consequences of making one’s genome publicly available are diverse. As HeLa 2.0 reveals, understanding of identifiability differs according to researcher, institution, and individual, and with open data models, refraining from pursuing re-identification of the participant is completely left to the ‘researcher’, whereas the public availability of data is often associated with ‘users,’ rather than only responsible researchers.

Post-identifiability is embedded in conflicting practices and expectations, from “open-controlled genomic databases” such as the European Genome Archive (ega-archive.org) to data altruism, whereby individuals would be able to make their data available for re-use for the common good, for example, for scientific research or to benefit public services, voluntarily and without financial reward (European Commission 2020). While providing data is framed as an altruistic act, new responsibilities, rights, rules, and actors are created with the regulation, where the aim is to “attain a higher level of trust in these services, without unnecessarily restricting these activities, and help develop an internal market for the exchange of such data” and “flexibility of application” (European Commission 2020). Data altruism opens up numerous issues regarding an individual’s genomic data. While such as a system may motivate participants to provide data to a centralized infrastructure rather than publishing genomic data directly on the internet without appealing to organizations offering data protection guarantees, it also begs the question: How could engagement take place in such systems? What would be the role of research ethics committees? What are the responsibilities of different stakeholders?

## Conclusion

The HeLa 2.0 controversy was not due to a few researchers’ practices or a failure of the peer-review system and ethics bodies, but rather it is a systematic issue, where genomic data and its peculiarity are at the core, as Skloot (2013) has suggested. It is often expected that social scientists, ELSI scholars, individual researchers, biobanks, patient organizations, and other stakeholders come up with fit-for-purpose solutions; however, as shown, identifiability as an issue is beyond an institution’s practices or the production of laws and guidelines. Data practices are not only involving a multiplicity of users and uses but also include certain meanings, values, and expectations that are social and systematic in nature and go beyond an individualizing or mainly technology-oriented approach. Consider for example, discussions around the FAIR principles, which also point to the need for fairness in data practices, taking the social, organizational, and institutional aspects into account. Against this background and building on the sociology of expectations perspective, we suggest looking back in order to go forward in reflecting the underlying concepts of privacy and identifiability. We believe that HeLa exceptionalism and the compartmentalization of the controversy solve one problem temporarily, whereas the post-genomic situation in which we find ourselves deserves a new understanding of post-identifiability and urgent societal discussion.

## Data Availability

The relevant documents and the online links that have been referenced within the article comprise the data as listed in Table 1 and described in the Methodology.

## Footnotes

1 The original press release (March 14, 2013) can be accessed in the form captured by the Web Archive at the following link: http://web.archive.org/web/20130314062014/http://www.embl.de/aboutus/communication_outreach/media_relations/2013/130311_Heidelberg/. The press release has been modified due to the controversy and can be accessed in the current form at the following link: https://www.embl.de/aboutus/communication_outreach/media_relations/2013/130311_Heidelberg/.

2 The original press release (March 17, 2013) can be accessed in the form captured by the Web Archive at the following link: https://web.archive.org/web/20210224172724/https://www.g3journal.org/content/suppl/2013/03/27/g3.113.005777.DC3/Landry_2013_EMBLStatement_Data.pdf.

3 The original press release (March 26, 2013) can be accessed in the form captured by the Web Archive at the following link: https://web.archive.org/web/20170813151734/http://www.g3journal.org/content/ggg/early/2013/03/26/g3.113.005777.full.pdf.

4 Database of Genotypes and Phenotypes (dbGaP) under National Center for Biotechnology Information (NCBI): https://www.ncbi.nlm.nih.gov/gap; NIH Advisory Committee to the Director HeLa Genome Data Access Working Group, on which two Lacks family members sit: https://acd.od.nih.gov/working-groups/hlgda.html.

5 The original blog post (March 26, 2013) can be accessed in the form captured by the Web Archive at the following link: https://web.archive.org/web/20130329085428/http://genomesunzipped.org/2013/03/henrietta-lackss-genome-sequence-has-been-publicly-available-for-years.php.

6 See the discussion between Yaniv Erlich, Bryan Howie and Joe Pickrell, at the latter’s blog (previous footnote).

7 Although we have included the *GDPR* as one of the analyzed documents, the *GDPR* was not in effect at the time of the controversy.
